# Matrix metalloproteinase-9 and -2 and tissue inhibitor of matrix metalloproteinase-2 in invasive pituitary adenomas: A systematic review and meta-analysis of case–control trials

**DOI:** 10.1097/MD.0000000000003904

**Published:** 2016-06-17

**Authors:** Hong-Yan Liu, Wei-Jun Gu, Cheng-Zhi Wang, Xiao-Jian Ji, Yi-Ming Mu

**Affiliations:** a Department of Endocrinology; b Department of rheumatology, Chinese PLA General Hospital, Haidian District, Beijing, P.R. China.

**Keywords:** invasion, meta-analysis, MMP-2, MMP-9, pituitary adenomas, TIMP-2

## Abstract

The extracellular matrix is important for tumor invasion and metastasis. Normal function of the extracellular matrix depends on the balance between matrix metalloproteinases (MMPs) and tissue inhibitors of metalloproteinases (TIMPs). The objective of this meta-analysis was to assess the relationship between expression of MMP-9, MMP-2, and TIMP-2 and invasion of pituitary adenomas.

We searched Pubmed, Embase, and the Chinese Biomedical Database up to October 2015. RevMan 5.1 software (Cochrane Collaboration, Copenhagen, Denmark) was used for statistical analysis. We calculated the standardized mean difference (SMD) for data expressed as mean ± standard deviation because of the difference in the detection method.

Twenty-four studies (1320 patients) were included. MMP-9 expression was higher in the patients with invasive pituitary adenomas (IPAs) than patients with noninvasive pituitary adenomas (NIPAs) with detection methods of IHC [odds ratio (OR) = 5.48, 95% confidence interval (CI) = 2.61–11.50, *P* < 0.00001), and reverse transcriptase-polymerase chain reaction (SMD = 2.28, 95% CI = 0.91–3.64, *P* = 0.001). MMP-2 expression was also increased in patients with IPAs at the protein level (OR = 3.58, 95% CI = 1.63–7.87, *P* = 0.001), and RNA level (SMD = 3.91, 95% CI = 1.52–6.29, P = 0.001). Meta-analysis showed that there was no difference in TIMP-2 expression between invasive and NIPAs at the protein level (OR = 0.38, 95% CI = 0.06–2.26, *P* = 0.29). MMP-9 expression in prolactinomas and nonfunctioning pituitary adenomas was also no difference (OR = 1.03, 95% CI = 0.48–2.20, *P* = 0.95).

The results indicated that MMP-9 and -2 may be correlated with invasiveness of pituitary adenomas, although their relationship with functional status of pituitary adenomas is still not clear. TIMP-2 expression in IPAs needs to be investigated further.

## Abstract 2

Supplemental Digital Content is available in the text

## 1 Introduction

Pituitary adenomas (PAs) are highly prevalent central nervous system tumors derived from adenohypophyseal cells. Despite the benign nature of PAs, they may invade adjacent structures, including the diaphragm sellae, suprasellar cistern, third ventricle, sellar floor, sphenoid sinus, dura mater, cavernous sinus, clivus, and others. Such invasive tumors were defined as invasive pituitary adenomas (IPAs) by Jefferson in 1940. According to this diagnostic criteria, about 45% of PAs have evidence of dural invasion.^[1]^ The therapeutic options include radical surgical resection, preoperative or postoperative radiotherapy, and medication, and except for prolactinomas, resection is the preferred option. It is, however, difficult for IPAs to be totally resected because of increased risk of cerebrospinal fluid leak and damage to cranial nerves and the internal carotid artery in the cavernous sinus.^[2]^ Furthermore, IPAs have a higher rate of recurrence, lower rate of remission, and poorer prognosis than noninvasive pituitary adenomas (NIPAs). Gültekin et al^[3]^ reported that the rate of recurrence (persistent and late recurrence) of invasive prolactinomas was 100% compared with 36% for noninvasive prolactinomas. To date, the pathogenesis of the invasion of PAs remains elusive.

Recent studies have reported the correlation between expression of matrix metalloproteinase (MMP)-9 and MMP-2 and IPAs. MMPs, also designated matrixins, are proteolytic enzymes containing a signal peptide, a propeptide, a catalytic domain, and a hemopexin domain^[4,5]^ capable of degrading the extracellular matrix, which is essential for tumor invasion.^[6]^ MMP-9 (gelatinase b) and MMP-2 (gelatinase a), classified as type IV collagenases, can degrade Collagen Types IV particularly,^[7]^ which is the prominent component of the basement membrane.^[8]^ Inasmuch as the basement membrane seems to play a critical role in tumor invasion,^[7]^ expression MMP-9 and -2 is conceived as an important sign of the tumor invasion. Previous studies have reported the relationship between MMP-9 and -2 expression and invasiveness of craniopharyngioma,^[9]^ medullary thyroid carcinoma,^[10]^ gastric carcinoma,^[11]^ ovarian serous tumor,^[12]^ glioma cells,^[13]^ et al. Recently, Ceylan et al reported that pituitary capsule, medial wall of the cavernous sinus, and reticular fiber roof of the hypophysis mainly consist of type IV collagen.^[14]^ Furthermore, Kawamoto et al found that type IV collagen is the key component of the dura mater, although its main compartment is type I collagen.^[15]^ These findings all demonstrate the significance of expression of MMP-9 and -2 to the invasion of PAs. Many studies have shown higher expression of MMP-9 and -2 in IPAs than in NIPAs. However, some authors have found either contrary results or no relationship between MMP-9 and -2 expression and invasion of PAs. We hypothesized that MMP-9 and -2 may be correlated with invasiveness of PAs, and act as critical biological markers in IPAs. However, considering the inconsistent conclusions of previous studies, we performed a meta-analysis of the literature to verify our hypothesis. The results of this meta-analysis contribute to learning about the pathogenesis of the invasion of PAs further and provide a novel therapeutic strategy for physicians. Besides, our results may be helpful for surgeons to make a decision on whether they could have an operation on patients, to assess the rate of remission and recurrence and to decide whether it is necessary to give an adjuvant therapy after surgery.

## 2 Materials and methods

### 2.1 Protocol

This meta-analysis of case–control trials was performed according to the MOOSE (Preferred Reporting Items for Systematic reviews and meta-analyses of Observational Studies) recommendations. This study was not a human or animal experiment, thus ethical approval was not necessary.

### 2.2 Search strategy

We conducted a search of Pubmed, Embase, and the Chinese Biomedical Database (all up to October 2015) for potentially eligible trials, without any language restriction. The subject headings and keywords we used were as following: “pituitary neoplasms,” “pituitary adenomas,” “pituitary adenoma,” “pituitary macroadenoma,” “pituitary tumor,” “prolactinoma,” “acromegaly,” “Cushing disease,” “Cushing's disease,” “Pituitary acth hypersecretion,” “matrix metalloproteinase 9,” “matrix metalloproteinase 2,” “gelatinase b,” “gelatinase a,” “type IV collagenase,” “MMP 9,” “MMP 2,” “MMP9,” “MMP2,” “MMP-9,” “MMP-2,” et al. A supplementary search of the reference lists from all retrieved trials and reviews was also performed. We contacted the corresponding author by mail if the articles were not available from databases.

### 2.3 Inclusion and exclusion criteria

Studies satisfying the following inclusion criteria were included: (1) case–control study design, (2) the detection method of MMP-9 and -2 expression were immunohistochemical staining (IHC) or (real-time) reverse transcriptase-polymerase chain reaction (RT-PCR), (3) results of IHC characterized by qualitative data and results of RT-PCR with average change and standard deviation (SD) were shown, and (4) diagnostic criteria for IPAs were met. Diagnostic criteria for IPAs were as following: (1) in modified Hardy's classification, grade III–IV adenomas or stage C–E tumors were defined as invasive.^[16]^ (2) In Knosp's classification, grade III–IV adenomas were defined as invasive.^[17]^ (3) Surgeons verified the penetration of sphenoid sinus or invasion of the parasellar nervus vasculairs. (4) Invasion of the diaphragm sellae, sellar bone, or surrounding endocranium was confirmed pathologically. (5) There was damage of surrounding structures according to magnetic resonance imaging and computed tomography. The PAs were considered invasive as long as they met one of the five diagnostic criteria.

### 2.4 Endpoints and data extraction

The primary endpoint was detection of MMP-9 expression using IHC at the protein level and RT-PCR at the mRNA level. The second endpoint was detection of MMP-2 expression using IHC and RT-PCR. The third endpoint was the expression of the tissue inhibitors of metalloproteinase (TIMP)-2 at the protein level. The final endpoint was microvessel density (MVD). Data including date of publication, name of first author, study type, detection methods, patient characteristics (mean age, age range, number of patients, and sex ratio), tumor type, and the aforementioned four endpoints were extracted from the eligible studies using a standard data-extraction form. Database search, eligibility evaluation, and data extraction were all performed independently by 2 authors (hongyan liu and weijun gu), with disagreements resolved by a third author.

### 2.5 Assessment of methodological quality

The Newcastle–Ottawa Scale (NOS) criteria (case–control study) was used for assessment of methodological quality, which contained the three following categories: (a) subject selection: (1) case definition was independently valid, (2) cases were consecutive or representative, (3) controls were from the community, and (4) controls did not have the same disease as the cases had. (b) Comparability of cases and controls: (1) controls were selected and analyzed according to the most important factor, and (2) controls were studied for a second important factor. (c) Exposure: (1) records were secure, (2) blind method was employed, (3) cases and controls had the same detection method, and (4) the two groups had a same nonresponse rate.^[18]^ The aforementioned items indicated a NOS score of 10, and a score of 5 or more was considered the inclusion criterion.

### 2.6 Statistical analysis

The Cochrane Collaboration's RevMan version 5.1 software was used for statistical analysis. Crude odds ratio (OR) with 95% confidence intervals (CIs) and standardized mean difference (SMD) with 95%CIs were used for qualitative and quantitative variables, respectively. SD was calculated according to the formula  
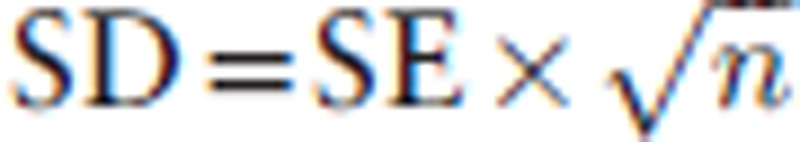
, if the data were expressed as mean ± SE (standard error of mean). We assessed the heterogeneity between trials with the method of Cochran's *Q*-statistic test and *I*^2^ test, measuring the extent of inconsistency—derived from heterogeneity rather than chance—in the results of eligible studies.^[19]^ A random-effects model was adopted on condition that *I*^2^ was more than 50% or *P* (*Q*-test) less than 0.05, otherwise, a fixed-effects model was used. Subgroup analysis of data expressed as mean ± SD was carried out according to detection methods to establish the derivation of heterogeneity. Sensitivity analysis by omitting any one study successively and funnel plots were conducted to assess the constancy of total estimate and publication bias, respectively. The drawing of forest plots and heterogeneity testing were completed in RevMan version 5.1 software.

## 3 Results

### 3.1 Study selection

**Figure 1:**
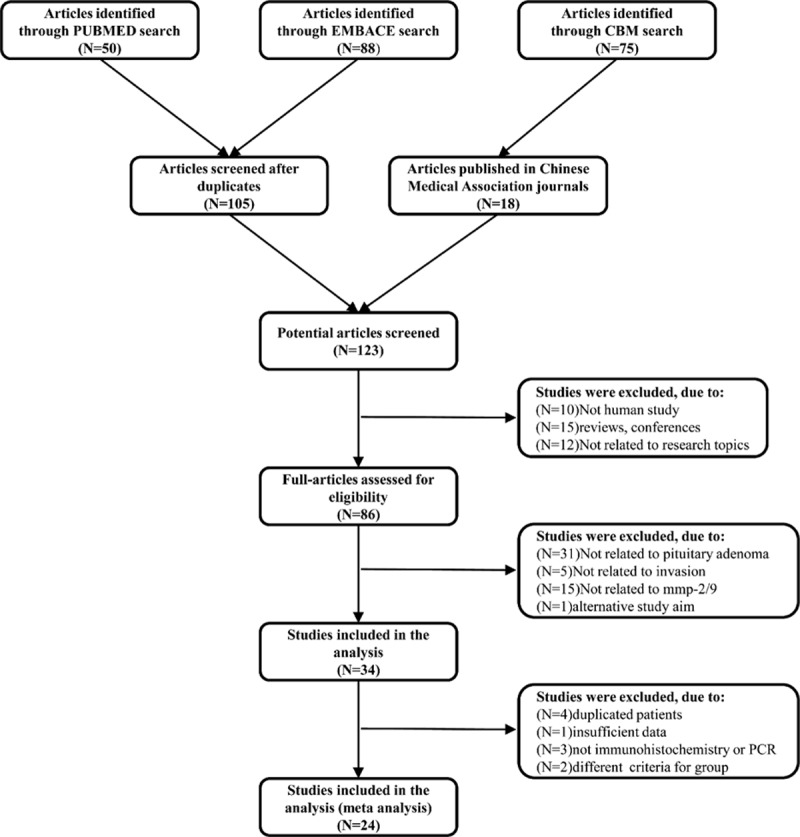
Flow chart of included studies.

We selected 213 candidate articles from our search, of which only 34 studies were regarded as eligibility. We discarded 10 studies for the following reasons: 4 inasmuch as they had duplicated subjects in other contained studies, 3 due to different detection methods [standard sandwich ELISA (enzyme linked immunosorbent assay) and Western blot], 2 in that some patients who met the diagnostic criteria for invasion, but were not included in the invasive group, and 1 owing to insufficient data. This resulted in a final total of 24 studies^[2,3,15,20–40]^ (Fig. 1).

### 3.2 Study characteristics

We outlined the characteristics of 24 studies included in our analysis (Table 1). The detection methods of IHC and RT-PCR were both used in seven studies. The IHC results of three studies were excluded because they were shown as mean ± SD and had a shortage of adequate data. Sixteen studies employed IHC only, and 7 adopted RT-PCR only. All included trials in the present study were of case–control design. The first study about the relationship between type IV collagenase expression and invasion of PAs was conducted by Kawamoto et al,^[15]^ thus the date of publications in the present study ranged from 1996 to 2015. There were a total of 1320 patients with PAs in the present meta-analysis. Two articles grouped the patients according to functional status of PAs rather than tumor invasion. The NOS score of methodological quality was summarized in Supplemental Table S1.

**Table 1:**
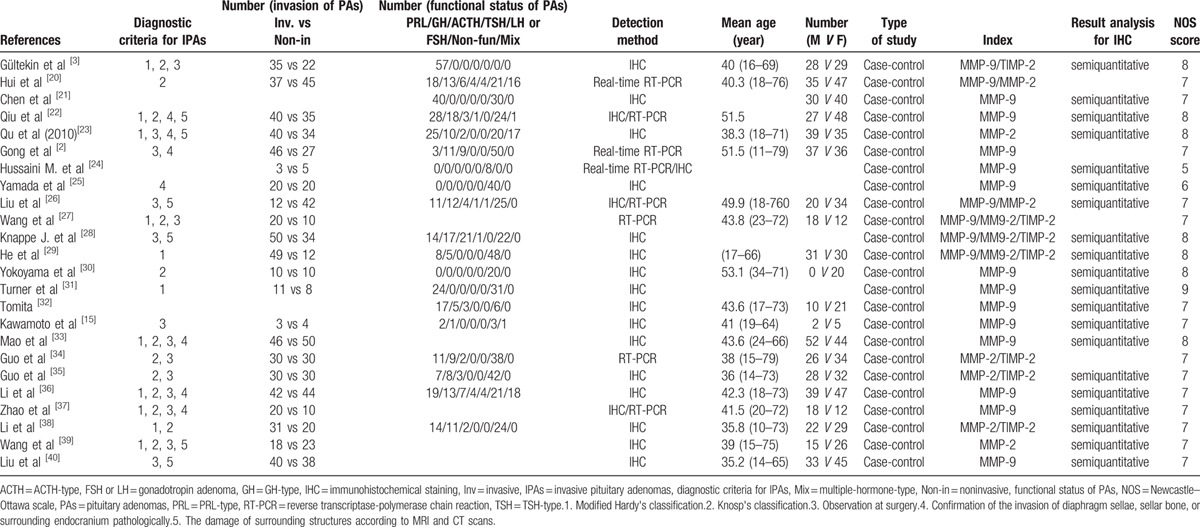
Characteristics of studies included.

### 3.3 Meta analysis

#### 3.3.1 Relationship of MMP-9 and -2 expression and invasion of pituitary adenomas

**Figure 2:**
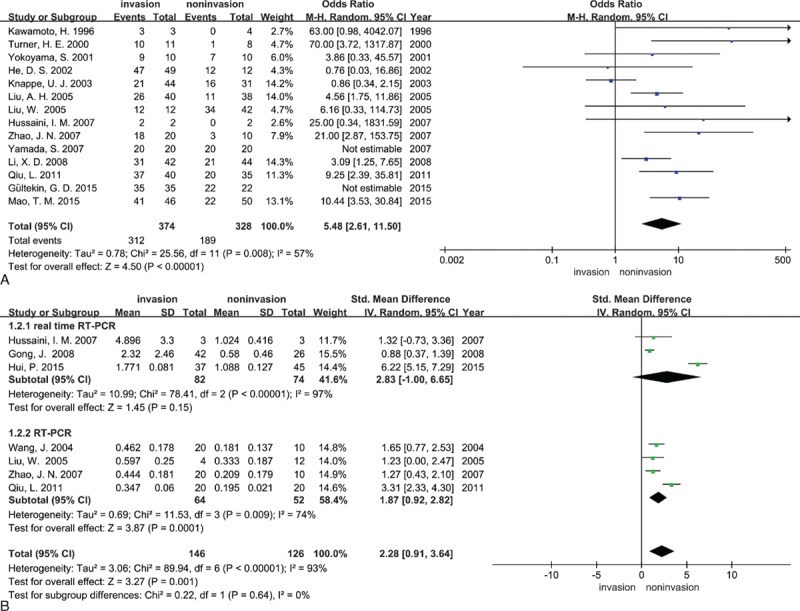
Forest plots for the relationship between MMP-9 expression and tumor invasiveness of PAs (A) at the protein level and (B) at the RNA level. M-H = Mantel–Haenszel test, IV = inverse variance, Random = a random effects model, CI = confidence intervals.

**Figure 3:**
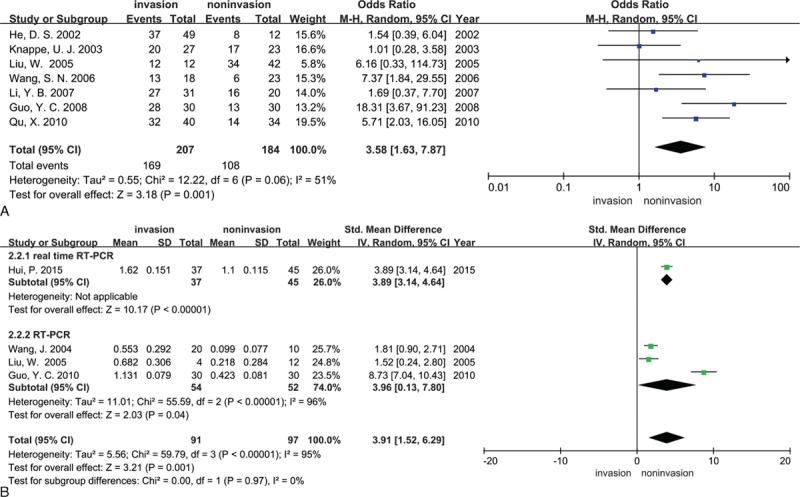
Forest plots for the relationship between MMP-2 expression and tumor invasiveness of PAs (A) at the protein level and (B) at the RNA level. M-H = Mantel–Haenszel test, IV = inverse variance, Random = a random effects model, CI = confidence intervals.

At the protein level or RNA level, there was substantial heterogeneity among the studies, making the use of a random effects model. Fourteen studies (374 IPAs and 328 NIPAs) and seven studies (146 IPAs and 126 NIPAs) showed MMP-9 expression at the protein level and RNA level, respectively. The results indicated that when we compared patients with invasive and NIPAs, MMP-9 expression was higher in the former with detection methods of IHC (OR = 5.48, 95% CI = 2.61–11.50, *P* < 0.00001; Fig. 2A), and RT-PCR (SMD = 2.28, 95% CI = 0.91–3.64, *P* = 0.001; Fig. 2B). Seven studies (207 IPAs and 184 NIPAs) and four studies (91 IPAs and 97 NIPAs) showed MMP-2 expression at the protein level and RNA level, respectively. MMP-2 expression was increased in patients with IPAs at the protein level (OR = 3.58, 95% CI = 1.63–7.87, *P* = 0.001; Fig. 3A), and RNA level (SMD = 3.91, 95% CI = 1.52–6.29, *P* = 0.001; Fig. 3B). The results of Sensitivity analysis indicated that no single study had a significant influence on the above four-pooled effect sizes (Supplemental Table S2–S5). Subgroup analytical results found that detection methods had no influence on pooled SMD of MMP-9 (test for subgroup differences: *I*^2^ = 0%, *P* = 0.64; Fig. 2B) and MMP-2 (*I*^2^ = 0%, *P* = 0.97; Fig. 3B) at the RNA level.

#### 3.3.2 Relationship of TIMP-2 expression and invasion of pituitary adenomas

**Figure 4:**
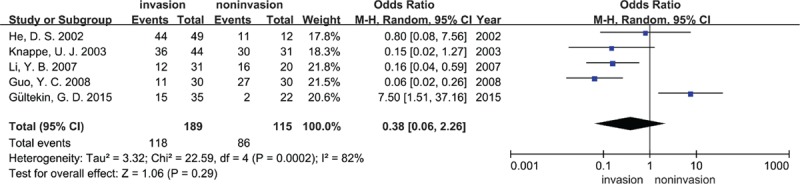
Forest plots for the relationship between TIMP-2 expression and tumor invasiveness of PAs at the protein level. M-H = Mantel–Haenszel test, Random = a random effects model, CI = confidence intervals.

Because of the considerable heterogeneity, the random effects model was also used for this meta-analysis. Five articles (189 IPAs and 115 NIPAs) reported TIMP-2 expression, but *P* = 0.29 (OR = 0.38, 95% CI = 0.06–2.26; Fig. 4) demonstrated that there was no statistical significance in TIMP-2 expression between IPAs and NIPAs. The results of sensitivity analysis showed that the study of Gültekin et al influenced the pooled OR greatly, indicating that this article may have been one of the sources of the substantial heterogeneity (Supplemental Table S6). Funnel plot were derived for MMP-9 expression in IPAs and NIPA at the protein and RNA levels and MMP-2 expression in IPAs and NIPA at the protein level. The symmetrical distribution funnel plot visually suggested that there was no publication bias (Figs. 6A and B, and 7), and the result of Begg's test also indicated no publication bias (data were not shown).

**Figure 5:**
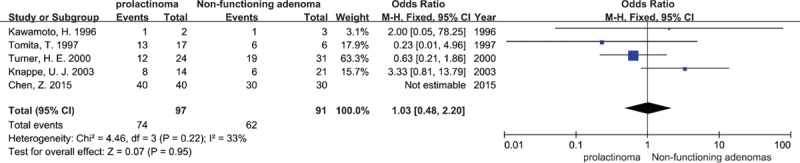
Forest plots for the relationship between MMP-9 expression and functional status of PA at the protein level. M-H = Mantel–Haenszel test, Fixed = a fixed effects model, CI = confidence intervals.

**Figure 6:**
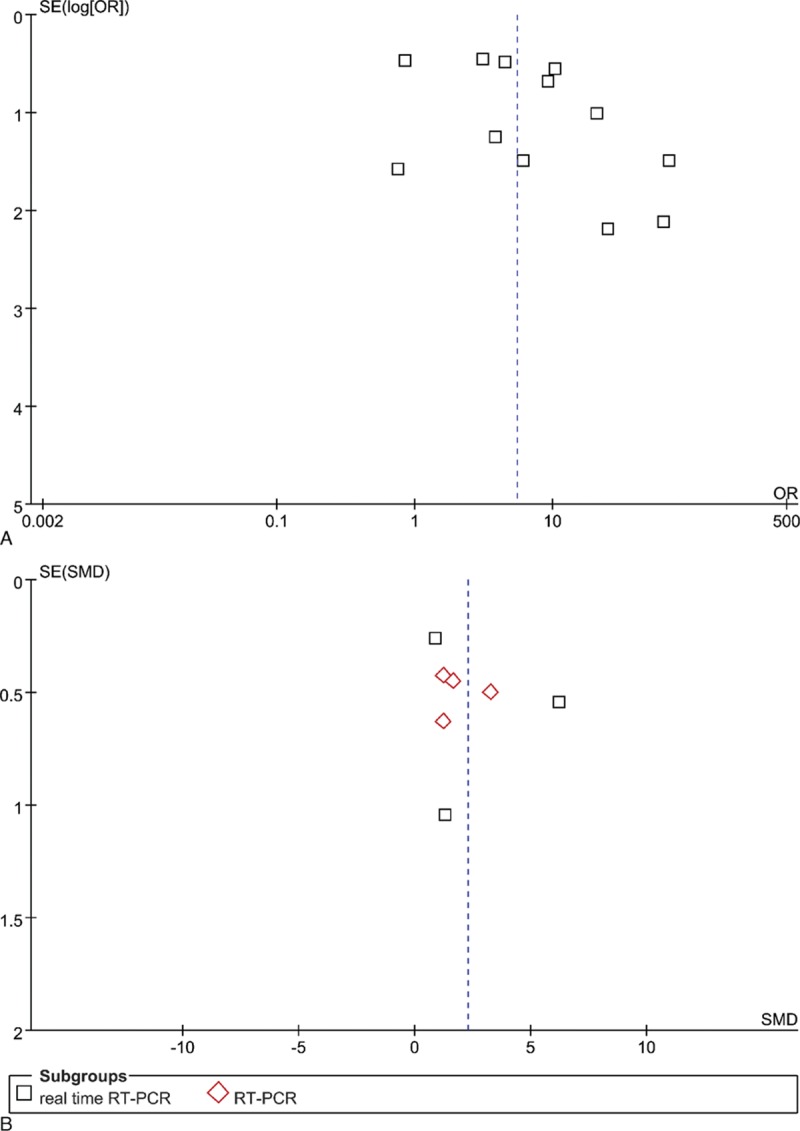
Funnel plots for the relationship between MMP-9 expression and tumor invasiveness of PAs. A. at the protein level. X and Y axes are OR and SE, respectively. B. at the RNA level. X and Y axes are SMD and SE, respectively.

**Figure 7:**
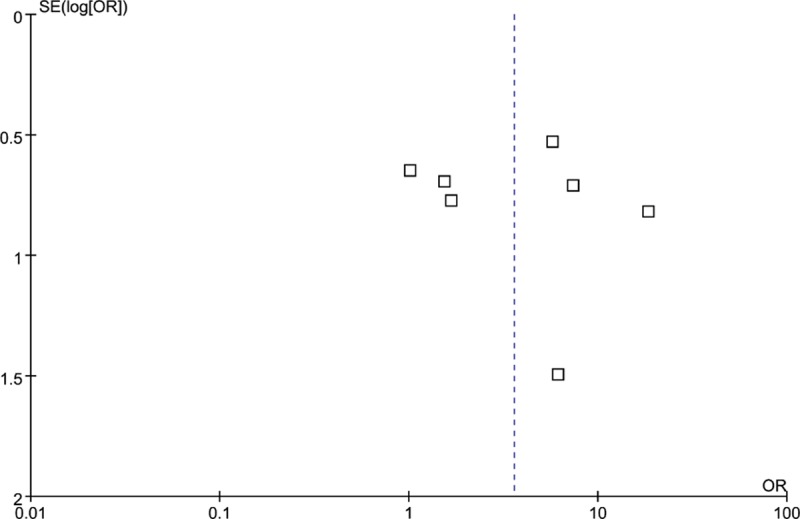
Funnel plots for the relationship between MMP-2 expression and tumor invasiveness of PAs at the protein level. X and Y axes are OR and SE, respectively.

#### 3.3.3 Relationship of MMP-9 expression and functional status of pituitary adenomas

MMP-9 expression in 97 prolactinomas and 91 nonfunctioning PAs was compared in five studies. There was no significant difference (OR = 1.03, 95% CI = 0.48–2.20, *P* = 0.95) between them using a fixed effects model (*I*^2^ = 33%, *P* = 0.22; Fig. 5).

#### 3.3.4 MMP-9 expression in primary and recurrent pituitary adenomas

Three studies analyzed the difference in MMP-9 expression between primary and recurrent adenomas (Supplemental Table S7), and the results showed a higher expression of MMP-9 in recurrent adenomas at the protein level (OR = 0.09, 95% CI = 0.01–0.53, *P* = 0.008) and at the RNA level (OR = −3.66, 95% CI = −5.15 to −2.17, *P* < 0.00001).

#### 3.3.5 Relationship between MMP-9 expression and microvessel density of pituitary adenomas

Two studies analyzed relationship between MMP-9 expression and MVD of PAs, but *P* = 0.09 (MD = 4.42, 95% CI = −0.66 to 9.50; Supplemental Table S8) demonstrated that there was no statistical significance between them.

## 4 Discussion

To the best of our knowledge, this is the first systematic review and meta-analysis to investigate the importance of type IV collagenases expression in IPAs. The NOS scores of studies included ranged from 5 to 9, which meet the requirement of meta-analysis. Our outcomes indicated that high expression of MMP-9 and -2 may be a significant cause of PAs invasiveness. As mentioned in the introduction, destruction of the basement membrane by MMP-9 and -2 may play a central role in the process of invasion of PAs. In 2006, Malik and Kakar^[41]^ found that pituitary tumor transforming gene facilitated tumor growth and metastasis through secretion of MMP-2 in vitro. One year later, Yoshida and Teramoto^[42]^ reported that increased expression of the discoidin domain receptor-1 promoted invasion of PAs via higher expression of MMP-9 and -2 in vitro. Recently, some proteins related to PAs invasion have been reported to work through regulating the level of MMP-9 and -2 in succession, such as reversion-inducing cysteine-rich protein with kazal motifs,^[43]^ FRα-targeted liposomal doxorubicin,^[44]^ and β-catenin.^[45]^ These all confirmed the central and critical role of MMP-9 and -2 in the invasion of PAs and were consistent with our results.

Pereda et al have suggested that high levels of MMPs (including MMP-9 and -2) stimulate pituitary cell proliferation and hormone secretion^[46]^ for the reason of growth factor anchored to extracellular matrix generated by MMPs.^[47]^ Meij et al found that macroadenomas (>40 mm) were 46% more likely to be invasive than microadenomas (≤10 mm).^[1]^ According to their ideas, the expression MMP-9 and -2 is related to size and functional status of PAs. Unfortunately, we found only four trials that analyzed the relationship between MMP-9 expression and tumor size, and their conclusions were inconsistent. We were unable to perform a meta-analysis because of the difference in detection methods, form of data expression and cutoff values for tumor size, and the small number of trials. This implies that more studies need to be conducted. Prolactinomas (account for 50%–55%) and nonfunctioning adenomas (account for 20%–25%) were the two most common types of PAs. Therefore, we carried out a meta-analysis to compare MMP-9 expression in the two different types of tumor, but we failed to find any difference. Given the limited number of eligible studies, we could not make a conclusion that the level of MMP-9 had nothing to do with the functional status of PAs.

Five studies included in this meta-analysis gave information about TIMP-2 expression in IPAs and NIPAs, and there was a nonsignificant decrease in IPAs statistically. If the study of Gültekin et al that had a great effect on pooled OR was removed, the decrease would have statistical significance. One study that was excluded from this meta-analysis because of the different detection method also demonstrated a decreased level of TIMP-2 in IPAs (*P* < 0.05).^[48]^ The TIMPs are endogenous MMP activity inhibitors that bind competitively to the binding sites for specific substrates of MMPs.^[49]^ To date, 4 homologous TIMPs (TIMP-1, -2, -3, and -4) are found in vertebrates.^[50]^ All MMPs, apart from MMP-14, -16, -19, and -24, can be inhibited by these four TIMPs.^[51]^ TIMP-2 seems to be an inhibitor of PAs invasion. However, TIMP-2 is unique because the complex of MT1-MMP/TIMP-2 acts as a pro-MMP-2 receptor and contributes to the activation of pro-MMP-2 on the cell surface.^[5,52]^ Furthermore, Valacca et al^[53]^ reported that the MT1-MMP/TIMP-2 complex is able to protect tumor cells against apoptosis by activating the AKP pathway in vitro. Recently, Stetler-Stevenson et al found that TIMP-2 can bind to a cell-surface signaling receptor, and then promote cell proliferation in an MMP-independent manner.^[54]^ At the same time, the level of TIMP-2 was found to be increased in many malignant tumors, which often indicates poor prognosis. These findings may explain the contrary result of Gültekin et al. However, more well-designed studies are required for meta-analysis of TIMP-2 expression in IPAs, and to determine the mechanism of action of TIMP-2 in tumor invasion.

Tumor angiogenesis, the case of which could be reflected through MVD of tumor, plays a critical role in the initiation and progression of tumor invasion and metastases.^[55]^ There have been studies reporting that MVD had a greater increase in IPAs compared with NIPAs (Supplemental Table S9). MMPs, especially MMP-9, could mediate tumor angiogenesis through mutual regulation and influence with vascular endothelial growth factor.^[55]^ According to the aforementioned, MVD of PAs may be correlated with MMP-9 expression. However, we found only two studies that investigated the relationship between them. And although our results suggested elevated MVD in MMP-9-positive PAs, there was no statistical significance, indicating that more studies need to be conducted. We also observed that the level of MMP-9 was higher in recurrent patients than that of primary patients, which means that patients with a high level of MMP-9 are likely to have poor prognosis.

There were several limitations to the present meta-analysis. First, given the relatively small number of studies included and overall sample size, the conclusion that MMP-9 and -2 acts as critical biological markers in IPAs should be treated with caution. Furthermore, the case–control study design was retrospective and had no rigorous quality control, and the grade of evidence was inferior to that of randomized controlled trials and cohort studies. Finally, despite sensitivity analysis and subgroup analysis conducted to search for the derivation of heterogeneity, we failed to minimize the high heterogeneity. Some potential reasons that resulted in great heterogeneity we thought are as following: Firstly, the data that we extracted were aggregated but individual, meaning that certain of baseline characteristics, such as age, sex, complications, and prognoses, were not taken into consideration. Secondly, although there are widely recognized diagnostic criteria of modified Hardy's classification and Knosp's classification for IPAs, making a definite diagnosis is still difficult because of unavoidable false negative and false positive results.^[56]^ Thirdly, there may be differences in detection methods of IHC among different laboratories.

In conclusion, the results of the present meta-analysis show that MMP-9 and -2 may be correlated with invasiveness of PAs, even though the study limitations mean our conclusions should be treated with caution. In consideration of the paucity of studies included, we cannot be certain of any differences in the level of MMP-9 among patients with PAs of diverse functional status and size. It is still unknown whether TIMP-2 acts as an enhancing or inhibitory factor in invasion of PAs, necessitating more large sample and well-designed studies.

## Acknowledgments

The authors are grateful to all the authors of the studies included in the present study and their study participants.

## Supplementary Material

Supplemental Digital Content
