# Supplementary material for: Matrix metalloproteinase-9 and -2 and tissue inhibitor of matrix metalloproteinase-2 in invasive pituitary adenomas: A systematic review and meta-analysis of case–control trials
Source: Medicine (Baltimore). 2016 Jun 17;95(24):e3904. doi: 10.1097/MD.0000000000003904 (PMC4998479; doi:10.1097/MD.0000000000003904)
Supplement: Supplemental Digital Content [file medi-95-e3904-s001.doc]

Table S1 The Newcastle-Ottawa Scale (NOS) score of methodological quality for studies included

| Study | NOS item* | | | | | | | | | | Total |
| --- | --- | --- | --- | --- | --- | --- | --- | --- | --- | --- | --- |
|  | Subject selection | | | | comparability | | exposure | | | |  |
|  | 1 | 2 | 3 | 4 | 5 | 6 | 7 | 8 | 9 | 10 |  |
| Gültekin, G. D. et al (2015)^3^ | 1 | 1 | 0 | 1 | 1 | 0 | 1 | 1 | 1 | 1 | 8 |
| Hui, P. et al (2015)^20^ | 1 | 1 | 0 | 1 | 1 | 0 | 1 | 0 | 1 | 1 | 7 |
| Chen, Z. et al (2015)^21^ | 1 | 1 | 0 | 1 | 1 | 0 | 1 | 0 | 1 | 1 | 7 |
| Qiu, L. et al (2011)^22^ | 1 | 1 | 0 | 1 | 1 | 1 | 1 | 0 | 1 | 1 | 8 |
| Qu, X. et al (2010)^23^ | 1 | 1 | 0 | 1 | 1 | 0 | 1 | 1 | 1 | 1 | 8 |
| Gong, J. et al (2008)^2^ | 1 | 1 | 0 | 1 | 1 | 0 | 1 | 0 | 1 | 1 | 7 |
| Hussaini, I. M. et al (2007)^24^ | 1 | 0 | 0 | 1 | 1 | 0 | 1 | 0 | 1 | 0 | 5 |
| Yamada, S. et al (2007)^25^ | 1 | 1 | 0 | 1 | 1 | 0 | 1 | 0 | 1 | 0 | 6 |
| Liu, W. et al (2005)^26^ | 1 | 1 | 0 | 1 | 1 | 0 | 1 | 0 | 1 | 1 | 7 |
| Wang, J. et al (2004)^27^ | 1 | 1 | 0 | 1 | 1 | 0 | 1 | 0 | 1 | 1 | 7 |
| Knappe, U. J. et al (2003)^28^ | 1 | 1 | 0 | 1 | 1 | 0 | 1 | 1 | 1 | 1 | 8 |
| He, D. S. et al (2002)^29^ | 1 | 1 | 0 | 1 | 1 | 0 | 1 | 1 | 1 | 1 | 8 |
| Yokoyama, S. et al (2001)^30^ | 1 | 1 | 0 | 1 | 1 | 1 | 1 | 0 | 1 | 1 | 8 |
| Turner, H. E. et al (2000)^31^ | 1 | 1 | 0 | 1 | 1 | 1 | 1 | 1 | 1 | 1 | 9 |
| Tomita, T. (1997)^32^ | 1 | 1 | 0 | 1 | 1 | 0 | 1 | 0 | 1 | 1 | 7 |
| Kawamoto, H. et al (1996)^15^ | 1 | 1 | 0 | 1 | 1 | 0 | 1 | 0 | 1 | 1 | 7 |
| Mao, T. M. et al (2015)^33^ | 1 | 1 | 0 | 1 | 1 | 1 | 1 | 0 | 1 | 1 | 8 |
| Guo, Y.C. et al (2010)^34^ | 1 | 1 | 0 | 1 | 1 | 0 | 1 | 0 | 1 | 1 | 7 |
| Guo, Y. C. et al (2008)^35^ | 1 | 1 | 0 | 1 | 1 | 0 | 1 | 0 | 1 | 1 | 7 |
| Li, X. D. et al (2008)^36^ | 1 | 1 | 0 | 1 | 1 | 0 | 1 | 0 | 1 | 1 | 7 |
| Zhao, J. N. et al (2007)^37^ | 1 | 1 | 0 | 1 | 1 | 0 | 1 | 0 | 1 | 1 | 7 |
| Li, Y.B. et al (2007)^38^ | 1 | 1 | 0 | 1 | 1 | 0 | 1 | 0 | 1 | 1 | 7 |
| Wang, S. N. et al (2006)^39^ | 1 | 1 | 0 | 1 | 1 | 0 | 1 | 0 | 1 | 1 | 7 |
| Liu, A. H. et al (2005)^40^ | 1 | 1 | 0 | 1 | 1 | 0 | 1 | 0 | 1 | 1 | 7 |

*NOS items: (1) independently valid case definition; (2) consecutive or representative cases; (3) community-derived controls; (4) controls having no disease the cases have; (5) controls selected according

to the most important factor; (6) controls selected according to a second important factor; (7) secure record; (8) blind method; (9) the same detection method for cases and controls; (10) a same non-response^18^

rate for cases and controls. The score of “1” means reported and adequate and “0” mean not reported or inadequate for individual item. The total ideal score is 10.

Table S2 Sensitivity Analyses of MMP-9 expression at protein level

| Excluded trial | No. of trials | No. of patients | Invasive group | Non-invasive group | OR(95% CI) | *P* value for OR | *I^2^*,% | *P* value for heterogeneity |
| --- | --- | --- | --- | --- | --- | --- | --- | --- |
| Kawamoto, H. 1996^15^ | 13 | 695 | 309 of 371 | 189 of 324 | 5.11 [2.43, 10.74] | ＜0.0001 | 58 | 0.008 |
| Turner, H. E. 2000^31^ | 13 | 683 | 302 0f 363 | 188 0f 320 | 4.76 [2.32, 9.78] | ＜0.0001 | 54 | 0.02 |
| Yokoyama, S. 2001^30^ | 13 | 682 | 303 of 364 | 182 of 318 | 5.69 [2.59, 12.53] | ＜0.0001 | 61 | 0.004 |
| He, D. S. 2002^29^ | 13 | 641 | 265 of 325 | 177 of 316 | 6.01 [2.81, 12.86] | ＜0.00001 | 59 | 0.007 |
| Knappe, U. J. 2003^28^ | 13 | 627 | 291 of 330 | 173 of 297 | 6.51 [3.82, 11.12] | ＜0.00001 | 13 | 0.32 |
| Liu, W. 2005^26^ | 13 | 648 | 300 of 362 | 155 of 286 | 5.52 [2.53, 12.03] | ＜0.0001 | 61 | 0.005 |
| Liu, A.H. 2005^40^ | 13 | 624 | 286 of 334 | 178 of 290 | 5.90 [2.46, 14.14] | ＜0.0001 | 61 | 0.004 |
| Yamada, S. 2007^25^ | 13 | 662 | 292 of 354 | 169 of 308 | 5.48 [2.61, 11.50] | ＜0.00001 | 57 | 0.008 |
| Zhao, J.N. 2007^37^ | 13 | 672 | 294 of 354 | 186 of 318 | 4.85 [2.28, 10.35] | ＜0.0001 | 56 | 0.01 |
| Hussaini, I. M. 2007^24^ | 13 | 698 | 310 of 372 | 189 of 326 | 5.29 [2.48, 11.29] | ＜0.0001 | 60 | 0.006 |
| Li, X.D. 2008^36^ | 13 | 616 | 281 of 332 | 168 of 284 | 6.27 [2.62, 15.01] | ＜0.0001 | 60 | 0.005 |
| Qiu, L. 2011^22^ | 13 | 627 | 275 of 334 | 169 of 293 | 5.20 [2.32, 11.67] | ＜0.0001 | 58 | 0.007 |
| Mao, T.M. 2015^33^ | 13 | 615 | 271 of 328 | 167 of 278 | 4.99 [2.24, 11.11] | ＜0.0001 | 55 | 0.01 |
| Gültekin, G. D. 2015^3^ | 13 | 645 | 277 of 339 | 167 of 306 | 5.48 [2.61, 11.50] | ＜0.00001 | 57 | 0.008 |

CI: confidence interval; OR: odds ratio

Table S3 Sensitivity Analyses of MMP-9 expression at RNA level

| Excluded trial | No. of trials | No. of patients | Invasive group  (No.) | Non-invasive group  (No.) | SMD(95% CI) | *P* value for SMD | *I^2^*,% | *P* value for heterogeneity |
| --- | --- | --- | --- | --- | --- | --- | --- | --- |
| Hussaini, I. M. 2007^24^ | 6 | 266 | 143 | 123 | 2.41 [0.92, 3.89] | 0.001 | 94 | ＜0.00001 |
| Gong, J. 2008^2^ | 6 | 204 | 104 | 100 | 2.53 [0.93, 4.13] | 0.002 | 92 | ＜0.00001 |
| Hui, p. 2015^20^ | 6 | 190 | 109 | 81 | 1.60 [0.85, 2.35] | ＜0.0001 | 74 | 0.002 |
| Wang, J. 2004^27^ | 6 | 242 | 126 | 116 | 2.39 [0.74, 4.03] | 0.004 | 94 | ＜0.00001 |
| Liu, W. 2005^26^ | 6 | 256 | 142 | 114 | 2.45 [0.90, 4.00] | 0,002 | 94 | ＜0.00001 |
| zhao, J. N. 2007^37^ | 6 | 242 | 126 | 116 | 2.45 [0.81, 4.10] | 0.003 | 94 | ＜0.00001 |
| Qiu, L. 2011^22^ | 6 | 232 | 126 | 106 | 2.10 [0.58, 3.63] | 0.007 | 94 | ＜0.00001 |

CI: confidence interva; SMD: standardized mean difference

Table S4 Sensitivity Analyses of MMP-2 expression at protein level

| Excluded trial | No. of trials | No. of patients | Invasive group | Non-invasive group | OR(95% CI) | *P* value for OR | *I^2^*,% | *P* value for heterogeneity |
| --- | --- | --- | --- | --- | --- | --- | --- | --- |
| He, D. S. 2002^29^ | 6 | 330 | 132 of 158 | 100 of 172 | 4.19 [1.74, 10.08] | 0.001 | 53 | 0.06 |
| Knappe, U. J. 2003^28^ | 6 | 341 | 149 0f 180 | 91 0f 161 | 4.60 [2.17, 9.75] | ＜0.0001 | 35 | 0.18 |
| Liu, W. 2005^26^ | 6 | 337 | 157 of 195 | 74 of 142 | 3.47 [1.48, 8.13] | 0.004 | 59 | 0.03 |
| Wang, S.N. 2006^39^ | 6 | 350 | 156 of 189 | 102 of 161 | 3.16 [1.29, 7.72] | 0.01 | 55 | 0.05 |
| Li, Y.B. 2007^38^ | 6 | 340 | 142 of 176 | 92 of 164 | 4.07 [1.68, 9.89] | 0.002 | 55 | 0.05 |
| Guo, Y.C. 2008^35^ | 6 | 331 | 141 of 177 | 95 of 154 | 2.81 [1.37, 5.76] | 0.005 | 34 | 0.18 |
| Qu, X. 2010^23^ | 6 | 317 | 137 of 167 | 94 of 150 | 3.25 [1.25, 8.42] | 0.02 | 55 | 0.05 |

CI: confidence interval; OR: odds ratio

Table S5 Sensitivity Analyses of MMP-2 expression at RNA level

| Excluded trial | No. of trials | No. of patients | Invasive group  (No.) | Non-invasive group  (No.) | SMD(95% CI) | *P* value for SMD | *I^2^*,% | *P* value for heterogeneity |
| --- | --- | --- | --- | --- | --- | --- | --- | --- |
| Hui, P. 2015^20^ | 3 | 106 | 54 | 52 | 3.96 [0.13, 7.80] | 0.04 | 96 | ＜0.00001 |
| Wang, J. 2004^27^ | 3 | 158 | 71 | 87 | 4.66 [1.39, 7.92] | 0.005 | 95 | ＜0.00001 |
| Liu, W.^26^ | 3 | 172 | 87 | 85 | 4.71 [1.72, 7.70] | 0.002 | 96 | ＜0.00001 |
| Guo, Y. C. 2010^34^ | 3 | 128 | 61 | 67 | 2.45 [0.87, 4.04] | 0.002 | 88 | 0.0002 |

CI: confidence interva; SMD: standardized mean difference

Table S6 Sensitivity Analyses of TIMP-2 expression at protein level

| Excluded trial | No. of trials | No. of patients | Invasive group | Non-invasive group | OR(95% CI) | *P* value for OR | *I^2^*,% | *P* value for heterogeneity |
| --- | --- | --- | --- | --- | --- | --- | --- | --- |
| He, D. S. 2002^29^ | 4 | 243 | 74 of 140 | 75 of 103 | 0.33 [0.04, 2.74] | 0.30 | 86 | ＜0.0001 |
| Knappe, U. J. 2003^28^ | 4 | 229 | 82 of 145 | 56 of 84 | 0.47 [0.06, 4.08] | 0.50 | 86 | ＜0.0001 |
| Li, Y. B. 2007^38^ | 4 | 253 | 106 of 158 | 70 of 95 | 0.49 [0.05, 5.19] | 0.55 | 85 | 0.0001 |
| Guo, Y. C. 2008^35^ | 4 | 244 | 107 of 159 | 59 of 85 | 0.62 [0.08, 4.61] | 0.64 | 80 | 0.002 |
| Gültekin, G. D. 2015^3^ | 4 | 247 | 103 of 154 | 84 of 93 | 0.15 [0.06, 0.36] | ＜0.0001 | 15 | 0.32 |

CI: confidence interval; OR: odds ratio

Table S7A MMP-9 expression in primary and recurrent pituitary adenomas at the protein level.

| study | Primary | | Recurrent | | *X*^2^ value | P value for *X*^2^ | OR(95% CI) |
| --- | --- | --- | --- | --- | --- | --- | --- |
|  | No. of patients | No. of positive | No. of patients | No. of positive |  |  |  |
| Turner, H. E. et al(2000)^31^ | 19 | 8 | 12 | 11 |  | 0.008 | 0.07 (0.01, 0.62) |
| Li, X. D. et al(2008)^36^ | 33 | 23 | 9 | 9 | 2.95 | ＜0.01 | 0.12 (0.01, 2.22) |
| Total (95% CI) | 52 |  | 21 |  |  |  | 0.09 (0.01, 0.53) |
|  |  | 31 |  | 20 |  |  |  |
|  | | | | | | | |
| Heterogeneity: P = 0.76; I² = 0% | | | | | | | |
| *P* value for total OR: 0.008 | | | | | | | |

CI: confidence interval; OR: odds ratio

Table S7B MMP-9 expression in primary and recurrent pituitary adenomas at the RNA level.

| study | Primary | | Recurrent | | t value | P value for t | MD(95% CI) |
| --- | --- | --- | --- | --- | --- | --- | --- |
|  | No. of patients | Mean ± SD | No. of patients | Mean ± SD |  |  |  |
| Gong, J. et al (2008**)**^2^ | 38 | 1.18 ± 4.01 | 7 | 4.84 ± 5.27 |  | ＜0.01 | -3.66 (-5.15, -2.17) |
|  | | | | | | | |
| P value for MD: < 0.00001 | | | | | | | |

CI: confidence interval; MD: mean difference

Table S8 Relationship between MMP-9 expression and microvessel density (MVD) of pituitary adenomas

| study | MMP-9 positive | | MMP-9 negative | | t value | P value for t | MD(95% CI) |
| --- | --- | --- | --- | --- | --- | --- | --- |
|  | No. of patients | Mean ± SD | No. of patients | Mean ± SD |  |  |  |
| Turner, H. E. et al(2000)^31^ | 6 | 7.656±0.634 | 7 | 5.561±1.942 |  | ＜0.05 | 2.09 (0.57, 3.62) |
| Liu, A. H. et al(2005)^40^ | 37 | 30.14±9.12 | 41 | 22.83±7.24 | 3.938 | ＜0.01 | 7.31 (3.63, 10.99) |
| Total (95% CI) | 43 |  | 48 |  |  |  | 4.42 (-0.66, 9.50) |
|  | | | | | | | |
| Heterogeneity: P = 0.01; I² = 85% | | | | | | | |
| P value for total MD: 0.09 | | | | | | | |

CI: confidence interval; MD: mean difference

Table S9 Relationship between invasion of pituitary adenomas and microvessel density (MVD)

| study | invasive | | noninvasive | | t value | P value for t |
| --- | --- | --- | --- | --- | --- | --- |
|  | No. of patients | Mean ± SD | No. of patients | Mean ± SD |  |  |
| Wang, S. N.et al(2006)^39^ | 18 | 47.65±15.61 | 23 | 18.34±8.49 | 8.419 | ＜0.001 |
| Liu, A. H. et al(2005)^40^ | 40 | 33.30±7,45 | 38 | 21.03±7.18 |  | ＜0.01 |
